# Clinical and biological risk factors associated with inflammation in patients with type 2 diabetes mellitus

**DOI:** 10.1186/s12902-021-00925-0

**Published:** 2022-01-06

**Authors:** Mohammed S. Ellulu, Hanen Samouda

**Affiliations:** 1 Department of Clinical Nutrition, Faculty of Applied Medical Sciences, Al-Azhar University – Gaza (AUG), Gaza, Palestine; 2 Luxembourg Institute of Health, Population Health Department, L-1445 Strassen, Luxembourg

**Keywords:** Diabetes, Inflammation, Adiponectin, Obesity, Body mass index, Cardiovascular diseases

## Abstract

**Background:**

Chronic inflammation has been associated with insulin resistance and related metabolic dysregulation, including type 2 diabetes mellitus (T2DM). Several non modifiable (i.e. genetic predisposition) and modifiable (i.e. sedentary lifestyle, energy-dense food) risk factors were suggested to explain the mechanisms involved in the development of inflammation, but are difficult to assess in clinical routine. The present study aimed to identify easy to asses clinical and biological risk factors associated with inflammation in patients with T2DM.

**Methods:**

One hundred nine patients (51 men, 58 women), 28–60 years old, from seven primary healthcare centers in Gaza City, Palestine, took part to the cross-sectional study (November 2013–May 2014). Study participants had T2DM with no history of inflammatory diseases, cardiovascular diseases, medication and/or any health condition that might affect the inflammatory markers, interleukin 6 (IL-6) and C-reactive protein (CRP). Inflammation was defined for IL-6 ≥ 2 pg/mL and CRP ≥ 6 mg/L. Multivariable logistic regressions were used to identify the relationship between inflammation and clinical and biological risk factors.

**Results:**

After adjustment for age and gender, inflammation seems to increase with increased body mass index (BMI) (OR: 1.427 [1.055–1.931]), increased fasting blood glucose (OR: 1.029 [1.007–1.052]) and decreased adiponectin values (OR: 0.571 [0.361–0.903]).

There were also significant relationships between inflammation and BMI (OR: 1.432 [1.042–1.968]), fasting blood glucose (OR: 1.029 [1.006–1.052]) and adiponectin (OR: 0.569 [0.359–0.902]), after adjustment for smoking habits and physical activity.

**Conclusion:**

Managing obesity and associated complications (i.e. hyperglycemia, high adiponectin levels) might help decreasing inflammation in individuals with T2DM.

**Supplementary Information:**

The online version contains supplementary material available at 10.1186/s12902-021-00925-0.

## Introduction

Type-2 diabetes mellitus (T2DM) is a chronic disease characterized by insulin resistance and insulin deficiency, triggering hyperglycemia or raised glucose levels in the blood (fasting blood glucose ≥126 mg/dL, postprandial glycaemia ≥200 mg/dL) [1, 2].

T2DM has been emphasized as a leading cause of depression, retinopathy, blindness, non-traumatic lower-limb amputation, neuropathy, renal failure, as well as a major cause of cardio- and cerebro-vascular diseases [3]. T2DM also affects the quality of life due to such as associated chronic complications [4]. Moreover, patients having T2DM are nearly twice as likely to die prematurely, compared to people free of type 2 diabetes. In particular, the number of deaths due to T2DM doubled worldwide, from 1990 to 2010 [5]. T2DM is also one of the 10 leading causes of death worldwide [6]. Unfortunately, about 463 million people live with diabetes worldwide, namely 9.3% of the global population in 2019, as reported in the 9th Edition of the Diabetes Atlas published by the International Diabetes Federation (IDF) [2]. 90% of people living with diabetes worldwide have T2DM [6]. In addition, 374 million adults worldwide have prediabetes. The number of individuals living with diabetes worldwide is expected to reach 578 million by 2030 and about 700 million by 2045 [6]. Several risk factors have been suggested to explain the mechanisms involved in the development of T2DM. Modifiable risk factors include the disappearance of traditional food habits in favor of the consumption of energy-dense food including more animal and processed foods, animal fat, trans fatty acids as well as a higher consumption of sugar-sweetened beverages, refined grains and/or polished rice characterized by a high glycemic index. Furthermore, a drastic shift from an active agrarian lifestyle to a high sedentary lifestyle, due to motorization and urbanization, significantly decreased physical activity and increased energy surplus and T2DM development [7–11]. Amongst the non-modifiable risk factors, genetic predisposition counts for 40 to 70% in the T2DM development, depending on the preexistence of the disease in one or two parents, although a considerable part of this heritability is due to obesity [12]. Actually, T2DM development is greatly influenced by the gene-environment interactions. In particular, genetic factors determine the development of both obesity and T2DM by influencing taste, food preferences and dietary patterns, sedentary lifestyle, as well as basal metabolism and calories burn [13]. Additional risk factors of T2DM include age, smoking, stress, sleep disorders and depression [14–16]. Further clinical risk factors associated with T2DM development include abdomino-visceral obesity, hyperglycemia, hypertension and dyslipidaemia, i.e. increased triglycerides and reduced high-density lipoprotein cholesterol (HDL-cholesterol) [17]. However, inflammation has been shown to be the main trigger of T2DM. This is done under the stimulus of the aforementioned risk factors and through the chronic activation of pro-inflammatory cytokine pathways in the tissues targeted by the insulin-related action such as the adipose tissue, the muscle mass and the liver [18–21]. Even a minimal glucose abnormality has been shown to be related to inflammatory processes and T2D complications [22]. Inflammation has also been linked with other conditions associated with T2DM, such as atherosclerosis and blood coagulation, metabolic syndrome, heart failure, cardiometabolic diseases, renal diseases and cancers [23–28].

Inflammation is the main cause of developing type 2 diabetes mellitus, yet inflammatory markers are rather not specific (i.e. C-Reactive Protein or CRP) or not usually measured in clinic (i.e. interleukin 6 or IL-6). Also, common risk factors of inflammation and T2DM (i.e. genetic predisposition, sedentary lifestyle, energy-dense food intake) are difficult to assess in clinical routine. Thus, the present study investigated, in a sample of patients with T2DM, the clinical risk factors associated with inflammation that might be easily measured in clinical routine.

## Participants and methods

Patients with type 2 diabetes mellitus (T2DM), defined by fasting glycaemia ≥126 mg/dL [2], and free from cardiovascular diseases, inflammatory diseases, medication and/or any health condition that might affect the inflammatory markers, interleukin 6 (IL-6) and C-reactive protein (CRP), were recruited from primary healthcare centers in Gaza City, Palestinian Territories. Seven centers were approached in Gaza City via cluster random sampling to obtain the data concerning the study. Medical history was investigated by the physicians. From the 484 screened patients, 109 individuals (51 men, 58 women), aged between 28 and 60 years, were eligible to participate in the study. All participants had a stable weight, or no fluctuation of more than 2% of their weight, for at least 2 months prior to the study. The enrollment of the participants in the study was performed between November 2013 and May 2014.

### Exclusion criteria include

- Pre-existing cardiovascular diseases: Hypertension, atherosclerosis, coagulation, open heart surgery, coronary artery disease and/or any other major adverse cardiac event,
- Inflammatory diseases: Autoimmune diseases,
- Any health condition that might affect the inflammatory markers, interleukin 6 and CRP: Allergies, asthma, malignancies, as well as liver, renal, respiratory, thyroid and/or acute infectious diseases,
- Using medication to treat cardiovascular and inflammatory conditions such as statin’s cholesterol-lowering agent, nonsteroidal anti-inflammatory drugs (NSAIDs) and cyclooxygenase-2 (COX-2) inhibitors, and corticosteroids as anti-inflammtory drugs.

### Data collection

The Case Report Form is reported in the Additional file 1.

#### Clinical and biological data

Age and gender were reported. Weight and height were measured. Body mass index (BMI) was calculated [BMI (kg/m^2^) = Weight (kg) / Height square (m^2^)] according to the World Health Organization (WHO) definition (2000) [29]. Seca 201 non-elastic tape was used to assess waist circumference (WC) at the level of the umbilicus and parallel to the floor, based on National Institute of Health (NIH) protocol (Lorenzo et al., 2007) [30]. Blood pressure (BP) was measured with the AccuSure® Mercury Sphygmomanometer. Fasting blood glucose (FBG) was measured with a glucose oxidase enzymatic colorimetric method. Total cholesterol (TC) and triglycerides (TG) concentrations were assessed with commercial ELISA kits. C-Reactive Protein (CRP) concentrations were measured with a CRP turbidimetric latex 1:5 kit. Interleukin 6 (IL-6) and adiponectin concentrations were measured with Sigma-Aldrich® ELISA kits via ELISA Reader.

#### Clinical and biological risk factors

Obesity was defined for BMI equal or more than 30 kg/m^2^ according to World Health Organization definition (2000) [29]. Abdominal obesity was defined for waist circumference equal or more than 102 cm for men and 88 cm for women according to National Institute of Health (NIH) protocol [30]. Hypertension was defined for systolic blood pressure (SBP) more than 140 mmHg and/or diastolic blood pressure (DBP) more than 90 mmHg according to the American Society of Hypertension and the International Society of Hypertension [31]. Hypercholesterolemia was defined for total cholesterol ≥200 mg/dL according to the National Cholesterol Education Program-Adult Treatment Panel III (NCEP-ATPIII) [32]. Hypertriglyceridemia was defined for triglyceride ≥150 mg/dL according to the NCEP-ATPIII [32]. Inflammation was defined for IL-6 ≥ 2 pg/mL and CRP ≥ 6 mg/L, according to American Heart Association [33].

#### Lifestyle habits

The *Global Physical Activity Questionnaire* (GPAQ) Version-2 [34] was used to assess physical activity at work, to travel and on recreational activities. *The Behavioral Risk Factor Surveillance System* (BRFSS) modified form was used to assess smoking habits [35]. The study participants were asked whether they were smokers at the present time; whether they have ever smoked; how long have they been quit smoking; how much did they smoke before stop smoking; and whether they are currently exposed to smoke (Additional file 1).

### Ethical considerations

All patients gave written informed consent before taking part to the study. All procedures were in accordance with the ethical standards and in line with the Helsinki Declaration of 1964, as revised in October 2008, in Seoul, Korea. Besides, the study was ethically approved by the Ethics Committee in Gaza (PHRC/HC/11/13) and the Ethical Committee of Universiti Putra Malaysia (JKEUPM), Ref Number FPSK_Mac (13) 04. Permission was obtained from the Director of Primary Healthcare Sector, Ministry of Health, Palestine.

### Statistical analysis

Data were analyzed by using the Statistical Package for Social Sciences version 21.0 software (SPSS Inc., Chicago, IL, USA). Descriptive statistics, including frequencies and percentages, were used to describe the categorical variables analyses. The central tendency of continuous variables was expressed in mean ± standard deviation (SD), minimal and maximal values. Univariate and multivariable logistic regressions were used to identify the clinical risk factors associated with inflammation. Odds Ratio (OR) were estimated. The multivariable models were adjusted on age, gender, smoking habits and/or physical activity. *P* values ≤0.05 were considered as statistically significant at the confidence level of 95%.

## Results

46.8% of men (*N* = 51) and 53.2% of women (*N* = 58) with non-insulin dependent T2DM, 28–60 years old, participated in this study. 78.0% of the participants had obesity and 88.1% had abdominal obesity. 34.9% of the participants had hypertension, 61.5% had hypercholesterolemia, 57.8% had hypertriglyceridemia and 11.9% had inflammation (high levels of CRP or IL-6). 19.3% of the participants were active smokers and 51.4% had low physical activity. The general characteristics of the population are presented in Table 1.

**Table 1:** General characteristics of the population (*N* = 109)

	N (%)	Mean ± SD	(95% CI)
Age (years)		49.65 ± 9.88	48.30–51.72
Men	51 (46.8%)		
Women	58 (53.2%)		
Obesity	85 (78.0%)		
Abdominal obesity	96 (88.1%)		
Hypertension	38 (34.9%)		
Hypercholesterolemia	67 (61.5%)		
Hypertriglyceridemia	63 (57.8%)		
Inflammation, hs-CRP ≥ 6 mg/L	42 (38.5%)		
Inflammation, IL-6 ≥ 2 pg/mL	23 (21.1%)		
**Anthropometry**			
BMI (kg/m^2^)		31.17 ± 7.33	30.74–33.04
WC (cm)		108.65 ± 15.24	106.15–111.21
SBP (mmHg)		127.06 ± 17.27	124.77–130.95
DBP (mmHg)		77.83 ± 9.24	77.15–80.57
FBG (mg/dL)		184.28 ± 59.79	169.91–198.64
TC (mg/dL)		185.03 ± 30.97	177.59–192.47
TG (mg/dL)		193.52 ± 106.3	167.89–219.06
Adiponectin (mg/L)		9.71 ± 0.43	8.85–10.57
hs-CRP (mg/L)		7.49 ± 7.10	5.17–8.71
IL-6 (pg/mL)		1.78 ± 0.63	1.63–1.93
**Lifestyle habits**			
Smoking habits (N, %)			
Active smokers	21 (19.3%)		
Past smokers	19 (17.4%)		
Passive smokers	22 (20.2%)		
No smokers	47 (43.1%)		
**Physical activity (N, %)**			
High	8 (7.3%)		
Moderate	45 (41.3%)		
Low	56 (51.4%)		

*Abbreviations*: *BMI* body mass index, *WC* waist circumference, *SBP* systolic blood pressure, *DBP* diastolic blood pressure, *FBG* fasting blood glucose, *TC* total cholesterol, *TG* triglyceride, *hs-CRP* high sensitivity C reactive protein, *IL-6* interleukin 6

### Clinical and biological correlates

Univariate analyses are detailed in Table 2. BMI (OR: 1.154 [1.039–1.282]), fasting blood glucose (OR: 1.011 [1.002–1.021]), triglycerides (OR: 1.005 [1.000–1.010]) and adiponectin (OR: 0.761 [0.615–0.942]) were associated with a higher risk to develop inflammation (Table 2).

**Table 2:** Univariate analyses. Relationship between inflammation and related clinical and biological risk factors

	Inflammation (hs-CRP ≥ 6 mg/L and IL-6 ≥ 2 pg/mL)			
	OR	95% CI		*P* value
		Min	Max	
Age (Years)	0.982	0.924	1.045	0.574
Gender (Men)	0.971	0.304	3.104	0.961
Gender (Women)	1.029	0.322	3.290	0.961
Smoking habits (Active)	0.719	0.133	3.899	0.702
Smoking habits (Past)	0.804	0.147	4.329	0.801
Smoking habits (Passive)	1.079	0.243	4.782	0.920
Smoking habits (Never)	–	–	–	0.971
Physical activity (Low)	1.000	0.106	9.393	1.000
Physical activity (Moderate)	0.875	0.088	8.660	0.909
Physical activity (High)	–	–	–	0.976
**BMI (kg/m**^**2**^**)**	**1.154**	**1.039**	**1.282**	0.008*
WC (cm)	1.031	0.986	1.078	0.177
SBP (mmHg)	1.011	0.977	1.047	0.524
DBP (mmHg)	1.200	0.955	1.088	0.556
**FBG (mg/dL)**	**1.011**	**1.002**	**1.021**	0.019*
TC (mg/dL)	0.966	0.979	1.013	0.623
**TG (mg/dL)**	**1.005**	**1.000**	**1.010**	0.045*
**Adiponectin (mg/L)**	**0.761**	**0.615**	**0.942**	0.012*

*Abbreviations*: *BMI* body mass index, *WC* waist circumference, *SBP* systolic blood pressure, *DBP* diastolic blood pressure, *FBG* fasting blood glucose, *TC* total cholesterol, *TG* triglyceride, *hs-CRP* high sensitivity C reactive protein, *IL-6* interleukin 6

*p values ≤0.05 were considered as significant

Multivariable analyses showed that inflammation is most likely to be associated with the increase of BMI (OR: 1.427 [1.055–1.931]) and fasting blood glucose values (OR: 1.029 [1.007–1.052]) and the decrease of adiponectin (OR: 0.571 [0.361–0.903]), after adjustment for age and gender.

After adjustment for age, gender, smoking and physical activity, high BMI (OR: 1.432 [1.042–1.968]) values, high fasting blood glucose concentrations (OR: 1.029 [1.006–1.052]), as well as low adiponectin (OR: 0.569 [0.359–0.902]) concentrations were associated with a higher risk of developing inflammation (Table 3).

**Table 3:** Multivariable logistic predictive models of inflammation (hs-CRP ≥ 6 mg/L and IL-6 ≥ 2 pg/mL) by clinical and biological risk factors

	Model 1				Model 2			
	OR	95% CI		*P* value	OR	95% CI		*P* value
		Min	Max			Min	Max	
**BMI (kg/m**^**2**^**)**	**1.427**	**1.055**	**1.931**	0.021*	**1.432**	**1.042**	**1.968**	0.027*
WC (cm)	0.928	0.821	1.049	0.235	0.928	0.820	1.050	0.237
SBP (mmHg)	1.012	0.912	1.123	0.817	1.014	0.913	1.126	0.797
DBP (mmHg)	0.942	0.780	1.139	0.538	0.938	0.771	1.141	0.520
**FBG (mg/dL)**	**1.029**	**1.007**	**1.052**	0.010*	**1.029**	**1.006**	**1.052**	0.013*
TC (mg/dL)	0.975	0.941	1.011	0.173	0.976	0.939	1.014	0.205
TG (mg/dL)	1.006	0.998	1.014	0.153	1.006	0.998	1.015	0.166
**Adiponectin (mg/L)**	**0.571**	**0.361**	**0.903**	0.017*	**0.569**	**0.359**	**0.902**	0.016*

Model 1: adjusted for age, gender

Model 2: adjusted for age, gender, smoking habits, physical activity

*Abbreviations*: *BMI* body mass index, *WC* waist circumference, *SBP* systolic blood pressure, *DBP* diastolic blood pressure, *FBG* fasting blood glucose, *TC* total cholesterol, *TG* triglyceride, *hs-CRP* high sensitivity C reactive protein, *IL-6* interleukin 6

*p values ≤0.05 were considered as significant

## Discussion

This study identified the clinical and biological risk factors associated with inflammation in a sample of patients with non-insulin dependent T2DM. Inflammation has been defined by the combination of CRP ≥ 6 mg/L and IL-6 ≥ 2 pg/mL. The percentage of patients who had inflammation was about 11.9%. After adjustment for age, gender, smoking habits and physical activity, higher BMI and fasting blood glucose values, and low adiponectin concentrations, were associated with a higher risk to develop inflammation.

### Effect of BMI on inflammation

**Fig. 1:**
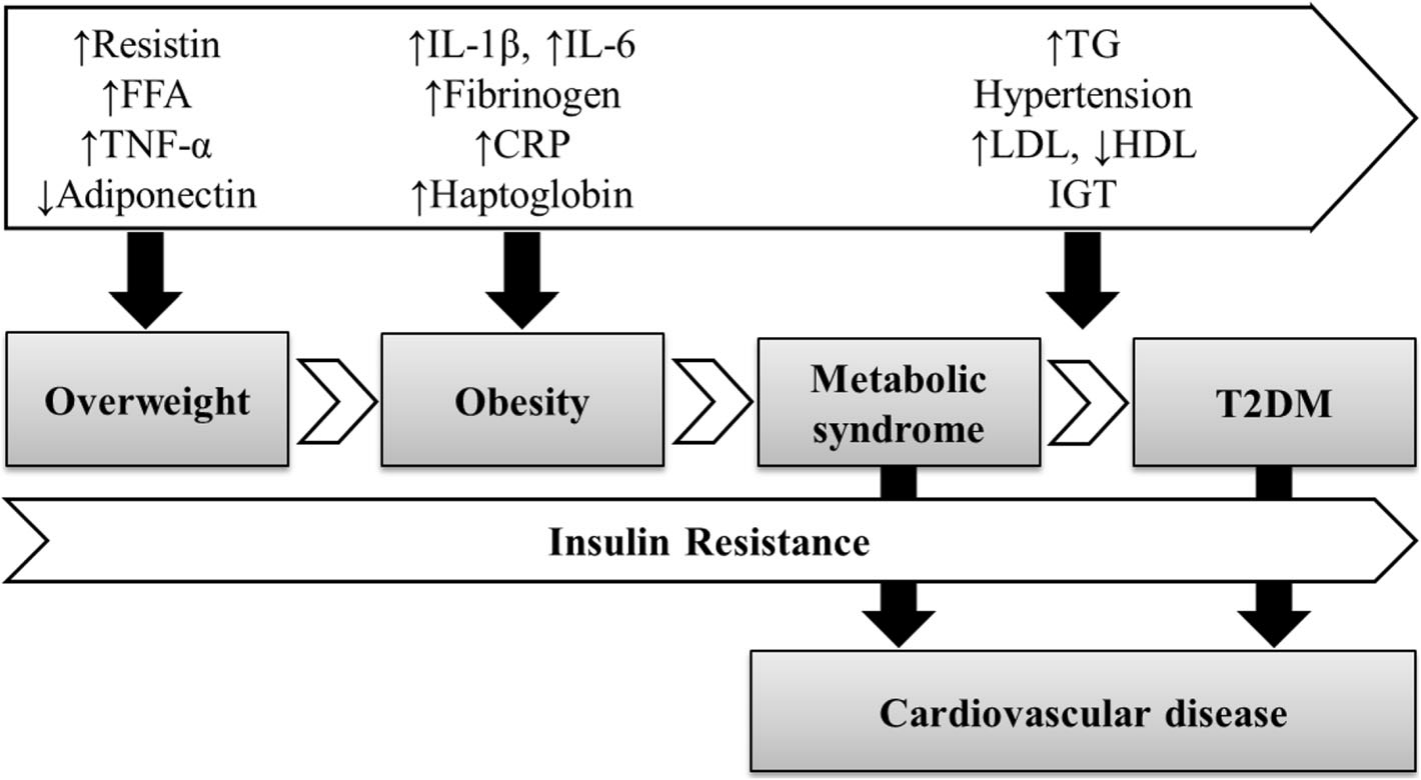
Relationship between inflammation, metabolically unhealthy obesity and development of type 2 diabetes and cardiovascular diseases (Ellulu, 2017). Abbreviations: FFA: free fatty acids. IGT: impaired glucose tolerance. HDL: high-density lipoprotein. LDL: low-density lipoprotein. T2D: type 2 diabetes

Obesity is a major cause of insulin resistance and associated metabolic dysregulation, including hypertension and dyslipidemia, which might trigger the development of type 2 diabetes [36]. Metabolically unhealthy obesity is a major contributor to the development of T2DM and cardiovascular diseases, possibly due to the oxidative stress and inflammation increase [24, 37, 38]. Insulin resistance has significantly been associated with obesity. This is possibly due to the release of bioactive metabolites such as free fatty acids, monocyte chemoattractant protein-1 (MCP-1) and pro-inflammatory cytokines by the adipocytes [39]. Figure 1 describes the relationship between inflammatory markers and disease occurrence, from metabolically unhealthy overweight and obesity to T2DM and cardiovascular diseases [40, 41]. Metabolically unhealthy obesity leads to the overexpression of CRP and IL-6, resulting in low-grade chronic inflammation [38]. A hyperplasia process might occur during the hypertrophy of the adipocytes as a response of the adipose tissue to over nutrition stimuli, inducing a permanent inflammatory state. Indeed, the enlarged adipocytes might reduce the blood supply to the fat cells and induce the hypoxia of the adipocytes [42]. This might consequently trigger the adipocytes necrosis as well as the infiltration of the macrophages in the adipose tissue, provoking the overproduction of pro-inflammatory mediators. This process induces the hyper-inflammation of the adipose tissue, which might results in a systemic inflammation and insulin-resistance, both possibly behind the development of obesity-related comorbidities [43]. In fact, the adipose tissue is not only a storage organ, but rather a metabolically dynamic organ, acting in interaction with the adipose, immune and endothelial cells functioning [44]. Three main inflammatory mediators are produced by the macrophages, the tumor necrosis factor-alpha (TNF-α), the interleukin-6 and the adiponectin [45]. The C-reactive protein is released by the hepatocytes, these latter being stimulated by the IL-6, which trigger long grade chronic inflammation [46]. The IL-6 are in particular released by certain pro-inflammatory serine kinases such as the inhibitor of nuclear factor kappa B (I_k_B) and c-JunN-terminal kinases, activated by the free fatty acids [47]. Several authors have investigated the significant relationship between obesity and inflammation. In particular, Straub et al. (2000) [48] highlighted that about one third of the total circulating IL-6 are released from the adipose tissue [49]. In fact, increased evidence has displayed that obesity constitutes a low-grade inflammatory state, possibly associated with metabolic dysregulation [50].

For instance, Dayal et al. (2014) [51] found that with each one unit of increment in BMI, CRP was more likely to increase by 37% (95% CI: 1.23–1.53, *P* < 0.001) amongst Indian children. Likewise, Klisic et al. (2014) [52] found that the level of CRP amongst post-menopausal women having obesity in Montenegro was higher than in post-menopausal women with normal weight (*P* < 0.001). Similarly, Kawamoto et al. (2013) [53] observed that BMI was the most significant predictor of inflammation, as assessed by CRP in community-dwelling persons. This was confirmed by several authors and in several populations. In particular, Warnberg et al. (2004) [54] showed high levels of CRP amongst Spanish adolescents having obesity, compared to the adolescents with normal weight as assessed by BMI (*P* < 0.05). Similar findings were observed regarding the relationship between BMI and IL-6 expression. In particular, Wannamethee et al. (2007) [55] showed increased IL-6 concentrations in 60–79 years old British men having obesity. Similarly, Rexrode et al. (2003) [56] confirmed the positive correlation between BMI and IL-6 amongst women who were free from cardiovascular diseases.

Likewise, Pradhan et al. (2001) [21] evaluated the level of inflammatory markers among US women having diabetes, compared to US women free of diabetes, through a prospective case-control study. The average BMI in women having diabetes was significantly higher (31.8 kg/m^2^) than in women free of diabetes (25.6 kg/m^2^). Similarly, the median of IL-6 in women having diabetes was about 2.0 pg/mL, compared to women free of diabetes (1.38 pg/mL). As well, the median of CRP in women having diabetes was significantly higher (0.69 mg/dL) than in women free of diabetes (0.26 mg/dL).

### Effect of adiponectin on inflammation

**Fig. 2:**
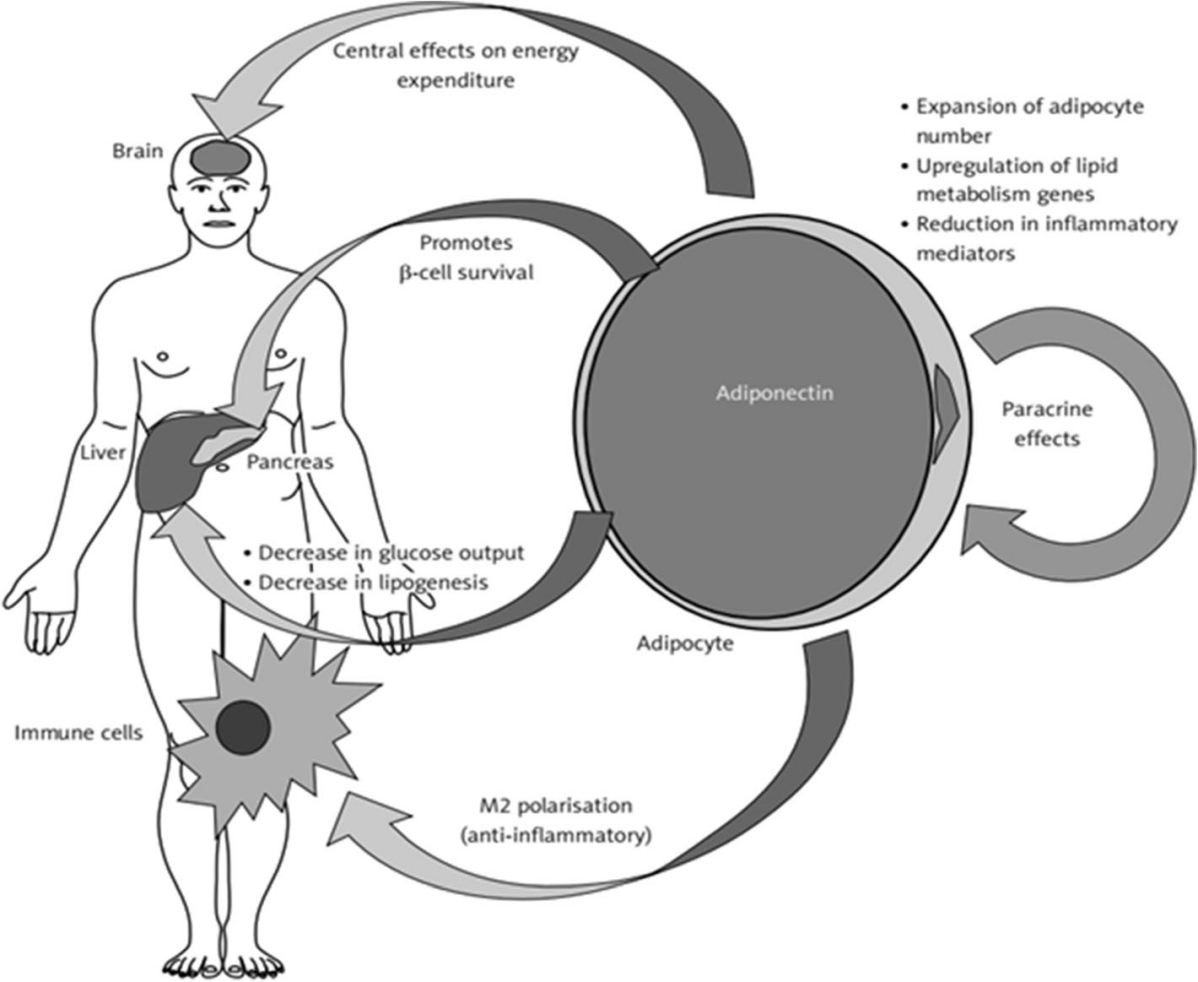
The role of Adiponectin on peripheral tissues insulin sensitivity (Ellulu et al., 2017)

Low adiponectin levels have been highlighted as having a significant impact on obesity occurrence, type 2 diabetes development and cardiovascular diseases raise, probably due to the adiponectin related insulin resistance and inflammation [57, 58]. Weight gain and obesity have in particular been associated with low serum levels of adiponectin [59]. Especially, the role played by the visceral adipocytes in releasing adiponectin has been emphasized [60]. Actually, increased serum level of inflammatory mediators such as IL-6 and TNF-α, released by the adipocytes, seem to inhibit the synthesis and secretion of adiponectin [60]. Conversely, in individuals with a metabolically healthy profile, adiponectin was associated with low inflammation levels, as well as a reduced risk of T2DM and atherosclerosis [60]. The role of high adiponectin levels in improving insulin resistance and immune system by its anti-inflammatory effect is described in Fig. 2**,** adapted from Ellulu et al. (2017) [40, 41]. The relationship between adiponectin and inflammatory markers has been highlighted in several studies. In particular, the hypoadiponectinemia status was previously significantly correlated with increased inflammation, as asessed by IL-6 [26]. Likewise, Hung et al. (2008) [61] highlighted a significant opposite relationship between adiponectin and inflammation as assessed by IL-6 and CRP, after adjustment for age, gender, waist to hip ratio and smoking habits.

### Effect of high fasting blood glucose levels on inflammation

Insulin resistance and β-cell exhaustion provoke an impaired glucose tolerance state, inducing the development of type 2 diabetes [62]. As shown in Fig. 1, inflammation triggers the insulin resistance, which might lead to T2DM raise [63]. Also, the relationship between inflammation and insulin resistance has been shown to be mediated by obesity [39]. Actually, inflammatory markers have been shown to be higher amongst individuals having high fasting blood glucose levels and metabolically unhealthy obesity [64]. Thus, the relationship between FBG and inflammatory markers in the present study is in accordance with the previously published studies. Lee et al. (2011) [65] showed a concomitant increase of CRP and FBG concentrations (*P* = 0.002), leading to cardiovascular events. Kawamoto et al. (2011) [64] observed significantly high CRP values related to fasting glucose in Japanese individuals displaying high levels of FBG (≥100 mg/dL) (*P* = 0.033), compared to Japanese individuals displaying low levels of FBG (< 100 mg/dL). This was also true amongst urban Portuguese adults, as well as in an adult population-based study in Germany, where CRP levels were significantly higher in individuals having elevated FBG levels, and were associated with metabolic syndrome occurrence [66, 67].

Similarly, Sarvottam and Yadav (2014) [26] identified that increased fasting blood glucose levels were significantly and positively correlated with inflammation as assessed by IL-6 concentrations, as well as with an increased endothelial dysfunction. Finally, Dandona et al. (2004) [68] highlighted that a high macronutrient intake, associated with obesity, was also significantly correlated with oxidative stress and inflammatory mediators (IL-6, TNF-α) increase.

### Limitation

A possible limitation of the present study might be due to its cross-sectional design. Therefore, we cannot conclude on any causality relationship in the interpretation of our findings.

## Conclusion

The present study highlighted increased BMI, high fasting blood glucose levels, as well as decreased adiponectin concentrations as clinical and biological risk factors of inflammation in a sample of patients with T2DM. Inflammation was defined for IL-6 ≥ 2 pg/mL and CRP ≥ 6 mg/L.

Tackling obesity and associated complications (i.e. hyperglycemia, high adiponectin levels) might help to decrease inflammation in individuals with T2DM.

### Recommendations for future research

We do recommend the measurement of body mass index and fasting blood glucose as risk factors of inflammation in patients with type 2 diabetes. Adiponectin might be difficult to measure in clinical routine. Glycosylated Hemoglobin (HbA1c) assessment should be considered, as a more sensitive biochemical marker than fasting blood glucose for type 2 diabetes diagnosis, when an appropriate funding is available to measure it.

## Supplementary Information

**Additional file 1.** Case Report Form. Case Report Form including the questionnaire on socio-demographics factors, medical history, smoking habits, physical activity pattern, physical examination and biochemical measures.

## Data Availability

The datasets used and/or analyzed during the current study are available upon request. The data requests should be addressed to Mohammed S. Ellulu.

## Abbreviations

BMI: Body mass index
BP: Blood pressure
BRFSS: The Behavioral Risk Factor Surveillance System
COX-2: Cyclooxygenase-2
CRP: C-reactive protein
DBP: Diastolic blood pressure
FBG: Fasting blood glucose
GPAQ: Global Physical Activity Questionnaire
HbA1c: Glycosylated Hemoglobin
HDL-cholesterol: High-density lipoprotein cholesterol
IDF: International Diabetes Federation
I_k_B: Inhibitor of nuclear factor kappa B
IL-6: Interleukin 6
MCP-1: Monocyte chemoattractant protein-1
NCEP-ATPIII: National Cholesterol Education Program-Adult Treatment Panel III
NIH: National Institute of Health
NSAIDs: Nonsteroidal anti-inflammatory drugs
OR: Odd-ratio
SBP: Systolic blood pressure
SD: Standard deviation
SPSS: Statistical Package for Social Sciences
TC: Total cholesterol
TG: Triglycerides
TNF-α: Tumor necrosis factor-alpha
T2DM: Type 2 diabetes mellitus
WHO: World Health Organization
WC: Waist circumference
