# Supplementary material for: Clinical and biological risk factors associated with inflammation in patients with type 2 diabetes mellitus
Source: BMC Endocr Disord. 2022 Jan 6;22:16. doi: 10.1186/s12902-021-00925-0 (PMC8740444; doi:10.1186/s12902-021-00925-0)
Supplement: Supplementary file 1 — Additional file 1. Case Report Form. Case Report Form including the questionnaire on socio-demographics factors, medical history, smoking habits, physical activity pattern, physical examination and biochemical measures. [file 12902_2021_925_MOESM1_ESM.docx]

**Case Report Form**

**Questionnaire**

**PART I: SOCIO-DEMOGRAPHIC FACTORS**

*Code*:

Q1- Sex: [ 1 ] Male [ 2 ] Female

Q2- Age in years: ........................................................

Q3- Marital status: [ 1 ] Single [ 2 ] Married

[ 3 ] Divorced [ 4 ] Widowed

Q4- Educational levels: [ 1 ] Secondary or less

[ 2 ] Diploma (2 years)

[ 3 ] Bachelor

[ 4 ] Master or Phd

Q5- Occupation: ..................................................................

Q6- Family size: ..................................................................

Q7- Menopause status:………………………...…………..

**PART II: MEDICAL HISTORY**

**Section A: *Parents’ medical history***

Q9- Do your parents have a chronic disease like hypertension or diabetes (the researcher will clarify the concept)?

1. Father:.......................... Type of disease:................................
2. Mother:......................... Type of disease:................................

**Section** **B: *Participant medical history***

Q10- Have you been suffering from any health problem at the present time?

[ 1 ] Yes [ 2 ] No

If yes; please specify? ....................................................................

Included: (Hypertensive, Diabetic, Hypertensive-diabetic).

Excluded: (Stroke, Renal, Hepatic,Respiratory, Thyroid, Arthritis).

Q11- At the present time, do you take any drug? (2 continued months).

[ 1 ] Yes [ 2 ] No

If yes; please specify?.....................................................................

Excluded: [ 1 ] Choletserol lowering agent (Statin).

[ 2 ] NSAIDs (Aspirin, Diclofen, Naproxen, COX-2 inhibitors).

[ 3 ] Anti-inflammatory corticosteroids

[ 4 ] Antibiotics.

[ 5 ] Insulin.

**PART III: SMOKING HABIT**

Q12- At the present time, are you smoker? (If No pass to Q13).

[ 1 ] Yes [ 2 ] No

Q13- Have you ever smoked ? (If No pass to Q16).

[ 1 ] Yes [ 2 ] No

Q14- How long have you been quit smoking? (......................................months).

Q15- How much did you smoke before stop smoking? (.....................cigarates).

Q16- Currently, are you exposed to smoke?

[ 1 ] Yes [ 2 ] No

| ***Interpretation of smoking habits*:**   1. Smokers. 2. Past smoker: one year stop smoking or more than 100 cigarettes in the past. 3. Passive smoker: one hour weekly closely contact with smoker person. 4. Non-smoker. |
| --- |

**PART IV: PHYSICAL ACTIVITY PATTERN**

| **No.** | **Questions** | **Response** | **Code** |
| --- | --- | --- | --- |
| ***Activity at work*** | | | |
| Q17 | Does your work involve vigorous-intensity activity that causes large increases in breathing or heart rate like for at least 10 minutes continuously? Ex:[carrying, lifting heavy loads, digging or construction work] | “YES” or “NO”  If “NO” skip to P4 | P1 |
| Q18 | In a typical week, on how many days do you do vigorous intensity activities as part of your work? | No. of days: .................... | P2 |

| **No.** | **Questions** | | **Response** | **Code** |
| --- | --- | --- | --- | --- |
| Q19 | How much time do you spend doing vigorous-intensity activities at work on a typical day? | | Hrs : Min  .........:......... | P3 |
| Q20 | Does your work involve moderate-intensity activity that causes small increases in breathing or heart rate such as brisk walking *[or carrying light loads*] for at least 10 minutes continuously? | | “YES” or “NO”  If “NO” skip to P7 | P4 |
| Q21 | In a typical week, on how many days do you do moderate intensity activities as part of your work? | | No. of days: .................... | P5 |
| Q22 | How much time do you spend doing moderate-intensity activities at work on a typical day? | | Hrs : Min  .........:......... | P6 |
| ***Travel to and from places*** | | | | |
| Q23 | Do you walk or use a bicycle for at least 10 minutes continuously to get to and from places? | | “YES” or “NO”  If “NO” skip to P10 | P7 |
| Q24 | In a typical week, on how many days do you walk or bicycle for at least 10 minutes continuously to get to and from places? | | No. of days: .................... | P8 |
| Q25 | How much time do you spend walking or bicycling for travel on a typical day? | | Hrs : Min  .........:......... | P9 |
| ***Recreational activities*** | | | | |
| Q26 | Do you do any vigorous-intensity sports, fitness or recreational (*leisure*) activities that cause large increases in breathing or heart rate like [*running or football,]* for at least 10 minutes continuously? | | “YES” or “NO”  If “NO” skip to P13 | P10 |
| Q27 | In a typical week, on how many days do you do vigorous intensity sports, fitness or recreational (*leisure*) activities? | | No. of days: .................... | P11 |
| Q28 | How much time do you spend doing vigorous-intensity sports, fitness or recreational activities on a typical day? | | Hrs : Min  .........:......... | P12 |
| Q29 | Do you do any moderate-intensity sports, fitness or recreational *(leisure*) activities that cause a small increase in breathing or heart rate such as brisk walking*,* (*cycling, swimming, and volleyball*) for at least 10minutes continuously? | | “YES” or “NO”  If “NO” skip to P16 | P13 |
| Q30 | In a typical week, on how many days do you do moderate-intensity sports, fitness or recreational (*leisure*) activities? | | No. of days: .................... | P14 |
| Q31 | How much time do you spend doing moderate-intensity sports, fitness or recreational (*leisure*) activities on a typical day? | | Hrs : Min  .........:......... | P15 |
| ***Sedentary behaviour*** | | | | |
| Q32 | How much time do you usually spend sitting or reclining on a typical day? | | Hrs : Min  .........:......... | P16 |
| Total Physical Activity (TPA-MET) =  [(P2*P3*8) + (P5*P6*4) + (P8*P9*4) + (P11*P12*8) + (P14*P15*4)]   - One MET is equivalent to a caloric consumption of 1kcal/kg/hour. - Moderate MET value of work, recreation, cycling or walking equal 4.0. - Vigorous MET value of work or recreation activities equal 8.0. | | | | |
| ***Conclusion of physical activity pattern*** | | | | |
| **High**: | | - If (P2+P11) ≥ 3 days & TPA-MET is ≥ 1500, or - If (P2+P5+P8+P14) ≥ 7 days & TPA-MET is ≥ 3000. | | |
| **Moderate**: | | - If it does not reach the criteria for high level of physical activity, or - If (P5+P8+P14) ≥ 5 days & [(P5+P6) + (P8*P9) + (P14*P15)] ≥ 150 minutes, or - If (P2+P5+P8+P11+P14) ≥ 5 days & TPA-MET ≥ 600. | | |
| **Low**: | | - If the level of physical activity does not reach the criteria for either high or moderate. | | |

**Physical Examination**

**PART I: PHYSICAL MEASUREMENTS**

**Section A: *Anthropometric measurements***

1. Hieght (cm) :.....................................................................
2. Weight (kg) :.....................................................................
3. Waist circumferance (cm) :.....................................................

**Section B: *Blood pressure***

1. Systolic (SBP) :..................................................... mmHg
2. Diastolic (DBP) :..................................................... mmHg

**PART II: BIOCHEMICAL MEASURES** (Pre-test for all participants**)**

| ***Biochemical measures*** | | ***Serum value*** |
| --- | --- | --- |
| C reactive protein (CRP) | mg/L |  |
| Interleukin 6 (IL-6) | pg/mL |  |
| Adiponectin | mg/L |  |
| Fasting blood glucose (FBG) | mg/dL |  |
| Total cholesterol (TC) | mg/dL |  |
| Triglyceride (TG) | mg/dL |  |
